# Protocol optimization and assessment of genotypic response for inbred line development through doubled haploid production in maize

**DOI:** 10.1186/s12870-023-04228-5

**Published:** 2023-04-26

**Authors:** Harjot Kaur, Mohammed Kyum, Surinder Sandhu, Gagandeep Singh, Priti Sharma

**Affiliations:** 1 Maize Section, Plant Breeding and Genetics, Punjab Agricultural University, Ludhiana, Punjab 141004 India; 2 Department of Agronomy, University of Florida, Gainesville, FL 32608 USA; 3 School of Agricultural Biotechnology, Punjab Agricultural University, Ludhiana, Punjab 141004 India

**Keywords:** Sub**-**tropical maize, Chromosomal doubling, Colchicine, Haploid induction rate, Doubled haploid

## Abstract

**Background:**

Doubled haploid technology offers the fastest route of inbred line development by rapidly fixing the desirable combinations in a single year. However, the differential response of haploid induction to genetic background of maternal lines accompanied with low induction rate and high mortality rate due to artificial chromosomal doubling of haploid seedlings creates hindrance in doubled haploid production on a commercial scale under tropical conditions. To speed up the hybrid breeding programme in sub-tropical maize, efforts are reported here to optimize the protocol for efficient production of fixed lines using haploid inducers. The second-generation haploid inducers i.e. CIM2GTAILs obtained from CIMMYT, Mexico were used for haploid induction in 13 F_1_s of diverse backgrounds. For standardization of chromosomal doubling protocol, various concentrations of colchicine and two seedling growth stages were used to determine the extent of chromosomal doubling and survival rate of doubled haploid plants.

**Results:**

A high mean haploid induction rate is obtained from CIM2GTAIL P2 (10%) as compared to CIM2GTAIL P1 (7.46%). Out of four treatments, CIMMYT reported protocol of chromosome doubling in tropical maize comprising combination of 0.07% colchicine and 0.1% DMSO at V_2_ stage is highly effective for acquiring doubled haploid plants in sub-tropical adapted maize with high survival rate of 52.7%. However, increasing the colchicine concentration from 0.07 to 0.1% led to high mortality rate.

**Conclusion:**

According to the findings, the haploid induction rate, survival rate and overall success rate varied depending upon the genotype of the inducer and the source population along with the concentrations of chemical used. The optimized protocol developed using CIMMYT haploid inducer CIM2GTAIL P2 for efficient doubled haploid production will not only fasten the breeding programme but will also reduce the production cost of doubled haploid with great efficiency in sub-tropical maize.

## Background

Doubled haploid (DH) breeding has revolutionized the existing field of plant breeding by serving as an efficient alternative method of inbred line development [5]. It offers the fastest route of inbred line development by rapidly fixing the desirable combinations of alleles in a single year. In maize, both in vitro and in vivo methods can be exploited to obtain haploids. The in vitro methods comprise the tissue culture approaches namely androgenesis and gynogenesis. On the other hand, the in vivo methods of haploid production include wide hybridization, CENH3 (Centromeric specific variant of histone H3) mediated approach, and the use of haploid inducer stock (HIS) [21]. However, the in vitro methods have proven to be less promising as compared to the in vivo methods of haploid induction (HI) in maize due to the ease of large-scale development of DH lines by the latter [16, 18]. Out of these, in vivo HIS-mediated haploid production is commercially viable because of its simplicity over the other methods.

The idea of *in planta* HI system emerged after the identification of a spontaneous mutant (Stock 6) in maize, capable of inducing 2–3% haploids [8]. This discovery led to the development of several maternal HISs using various breeding methodologies. The paternal and maternal HISs were derived from *ig1* mutant (Wisconsin-23) and Stock-6, respectively. However, the high haploid induction rate (HIR) of the maternal HISs made it more reliable for its utilization in breeding programs [19]. The mechanism of HI remained enigmatic until the genomic era, which hindered the understanding of its genetic potential and further transferring it into different genetic backgrounds.

The advent of molecular markers, gene mapping, and various molecular techniques helped in the identification of the genetic nature of HI. Lashermes and Beckert (1988) initially recognized the HI trait as quantitative that can be strengthened by selection [14]. The polygenic nature of HI was identified by quantitative trait loci (QTL) analyses, their fine mapping, and cloning [11]. These QTLs were then transferred into different genetic backgrounds using molecular markers for developing the modern genetic HISs such as RWK, RWS, CAUHOI, UH400, etc. [11, 12, 23]. These modern haploid inducer lines with high HIR (> 6%) have made the large-scale use of DH easy in the case of maize breeding.

The HISs showed varied response of HI in different genetic backgrounds (used as female parents). Moreover, the faithful doubling of chromosomes in haploids is another major limitation. To date, colchicine, N_2_O, and herbicides having pronamide, oryzalin, and amyprophos-methyl (APM) have been used for chromosomal doubling [1, 16]. The success rate of chromosomal doubling is affected by the duration of treatment, stage of the target tissue, and handling [16]. Optimization of methods for chromosomal doubling is required to attain efficiencies in DH generation pipelines since the chromosomal doubling is labor-intensive and costly [4]. Recently, Chaikam et al. [2] developed a novel method that involves treating the crown part of haploid seedlings and their roots at the V_2_ stage with the colchicine solution at varied concentrations. Their results displayed approximately 5.6 times more success rate than the standard methods, showing the immense potential to translate this technology at commercial maize DH program.

Until now, there was the unavailability of tropical HISs with high HIR as well as favorable agronomic performance in maize. The temperate inducer lines, UH400 and RWS, showed similar HIRs in the tropical conditions but were asynchronous to the tropical germplasm having weak plant vigor, limited seed set, and high susceptibility to diseases [20]. This hindered the maintenance of these lines under tropical conditions. To overcome these issues, CIMMYT generated first-generation tropically adapted lines (TAILs) that showed relatively better agronomic performance as compared to the temperate inducer lines. But these lines still lacked improvement in case of HIR, plant vigor and susceptibility to diseases in the tropics. Therefore, CIMMYT developed second-generation tropicalized haploid inducer lines (CIM2GTAILs) that have proven to show high HIR and comparatively better agronomic performance than the TAILs.

At Punjab Agricultural University (PAU), continuous efforts are being made to develop high-yielding maize hybrids for utilization by farmers. To accelerate the inbred line development program and to fasten the hybrid release process, optimization of DH technology and availability of high-frequency inducers would be of great help to the maize breeders. Earlier, the HISs had poor adaptation to tropical climates therefore PAU has acquired CIM2GTAILs, developed by CIMMYT. Efforts were made to analyze the HIR of CIM2GTAILs in different genetic backgrounds and protocol optimization for the efficient production of DH. The objective of this study is to optimize the protocol for DH production using haploid inducer lines viz. CIM2GTAIL P1 and CIM2GTAIL P2 and different strategies for the chromosomal doubling of haploids raised from high yielding and adapted lines of the region. The comparative HIR of each HIS, genotypic effect on inducer population for haploid production and the effect of different protocols for chromosomal doubling are reported in the present investigation.

## Results

### Inducer and genotype effect

**Fig. 1:**
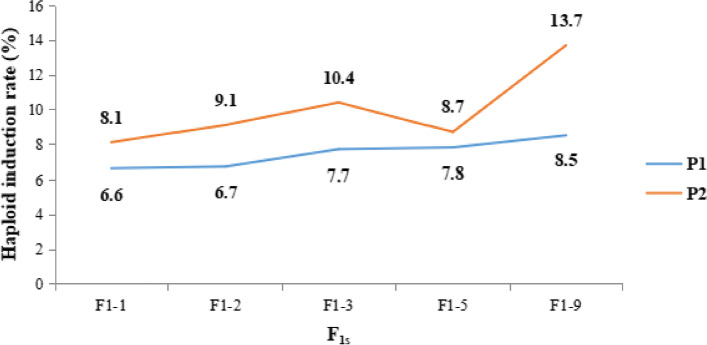
Comparative mean haploid induction rate obtained from inducer stock lines P1 and P2

In this study, 13 F_1_s from elite backgrounds were used to carry out the induction crosses with the inducer stocks in Spring and *Kharif* season (2021). Among the ten successful crosses conducted, the inducer P1 had a mean HIR of 7.46%, reaching 8.5% when crossed with F_1_-9 (Table 1), whereas the mean HIR of inducer P2 was 10%, with F_1_-9 recorded as 13.7% (Table 2). Overall, the experiment showed that the mean HIR of the CIM2GTAIL P2, in general was higher (10%) as compared to CIM2GTAIL P1 (7.46%) (Fig. 1).

**Table 1:** Seasonal data for haploid induction rate of different F_1_s crossed with CIM2GTAIL P1

	Spring 2021			*Kharif* 2021			
F_1_s	Total seed count	No. of haploid seeds	Haploid induction rate (%)	Total seed count	No. of haploid seeds	Haploid induction rate (%)	Mean HIR (both seasons)
F_1_-1	1000	68	6.8	1300	84	6.5	6.6
F_1_-2	525	36	6.8	500	34	6.6	6.7
F_1_-3	270	21	7.8	330	25	7.6	7.7
F_1_-5	515	39	7.6	400	32	8.0	7.8
F_1_-9	1500	127	8.5	2100	180	8.6	8.5
**Grand total**	3810	292	**Mean** = 7.5	4630	354	**Mean** = 7.46	**Mean** = 7.46

**Table 2:** Seasonal data for haploid induction rate of different F_1_s crossed with CIM2GTAIL P2

	Spring 2021			*Kharif* 2021			
F_1_s	Total seed count	No. of haploid seeds	Haploid induction rate (%)	Total seed count	No. of haploid seeds	Haploid induction rate (%)	Mean HIR (both seasons)
F_1_-1	2500	209	8.4	3100	246	7.9	8.1
F_1_-2	2300	215	9.3	2500	229	8.9	9.1
F_1_-3	5100	517	10.1	3900	423	10.8	10.4
F_1_-5	400	35	8.8	440	38	8.6	8.7
F_1_-9	1200	164	13.6	1100	153	13.9	13.7
**Grand total**	11,500	1141	**Mean** = 10.04	11,040	1082	**Mean** = 10.02	10.0

### Chromosomal doubling efficiency and calculation of survival rate

**Fig. 2:**
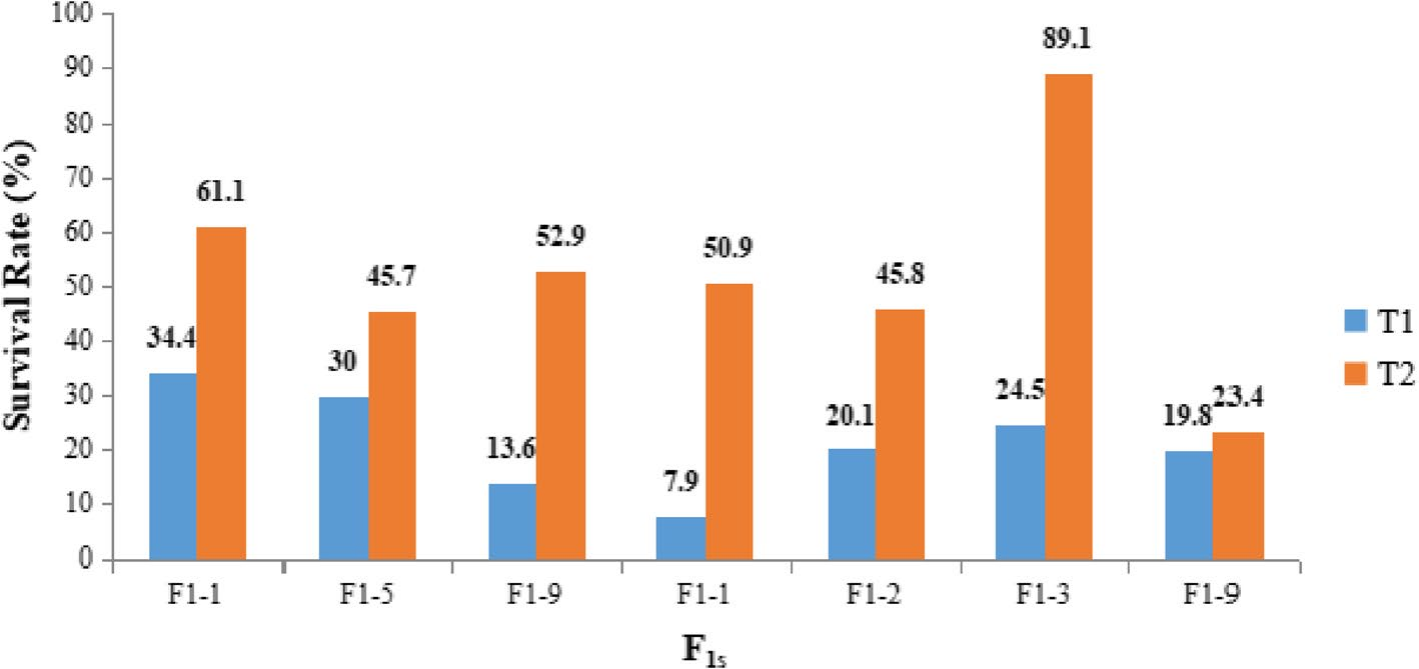
Comparative survival rate obtained from chromosomal doubling treatments (T1 and T2)

**Fig. 3:**
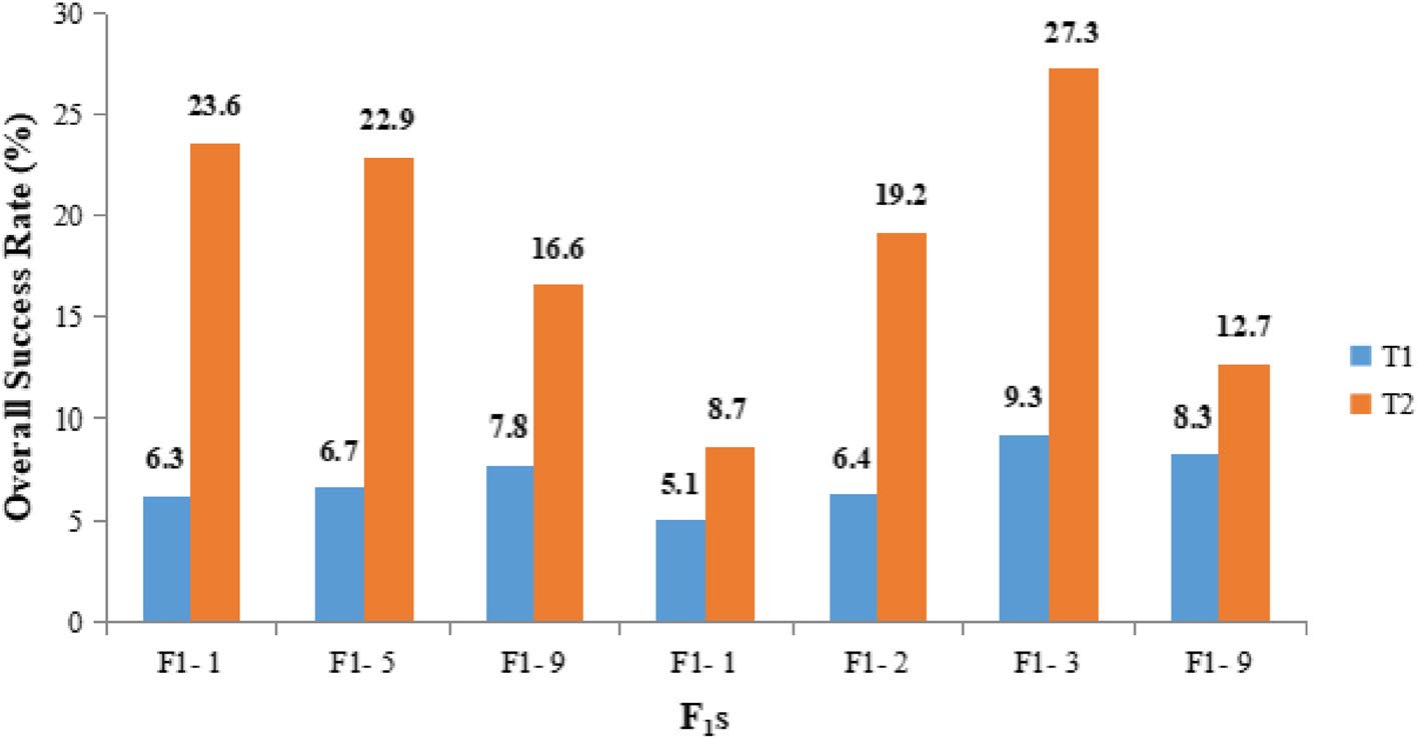
Comparative overall success rate obtained from chromosomal doubling treatments (T1 and T2)

Two different treatments performed among various crosses represented an elevated SR in the case of T2 as compared to the T1 method. The crosses generated with the inducer P2 have proven to be effective for an increase the SR of plants. The CIMMYT protocol developed by Chaikam et al. [2] was used for optimization. Three different combinations of the chemicals (Colchicine and DMSO) were used in the T2 method, of which 0.04% Colchicine with 0.5% DMSO and 0.07% Colchicine accompanied by 0.1% DMSO had a low mortality rate as compared to 0.1% Colchicine with 0.1% DMSO. According to the present study, 0.07% Colchicine and 0.1% DMSO are highly efficient for an elevated SR as well as doubling efficiency. It was also observed that the SR (Fig. 2) and OSR (Fig. 3) of the plants obtained from the different crosses varied depending on the genotype of the source population. The mean SR and OSR were found to be higher in T2 as compared to T1 (Table 3). The mean SR was 21.47% and 52.7% in T1 and T2, respectively whereas the mean OSR in T1 and T2 was 15.23% and 18.71%, respectively. Both were found to be higher in the case of T2.

**Table 3:** Comparative performance of chromosome doubling methods for seedling survival rate and overall success rate

F_1_s	T1				T2			
	No. of seeds germinated	No. of D_0_ plants survived	SR (%)	OSR (%)	No. of seeds germinated	No. of D_0_ plants survived	SR (%)	OSR (%)
**CIM2GTAIL P1**								
F_1_-1	64	22	34.4	6.3	72	44	61.1	23.6
F_1_-5	30	9	30.0	6.7	35	16	45.7	22.9
F_1_-9	103	14	13.6	7.8	157	83	52.9	16.6
**CIM2GTAIL P2**								
F_1_-1	215	17	7.90	5.1	220	112	50.9	8.7
F_1_-2	204	41	20.1	6.4	214	98	45.8	19.2
F_1_-3	400	98	24.5	9.3	450	401	89.1	27.3
F_1_-9	96	19	19.8	8.3	205	48	23.4	12.7
Mean			21.47	15.23	Mean		52.7	18.71

### Doubled haploid cobs obtained

The DH seeds (D_1_) (Table 4) obtained after selfing of fertile D_0_ plants are maintained in the Maize experimental field area of PAU, Ludhiana.

**Table 4:** D_1_ cobs obtained after selfing of D_0_ plants

F_1_s	No. of D_1_ cobs obtained (T1)	No. of D_1_ cobs obtained (T2)
**CIM2GTAIL P2**		
F_1_- 1	11	19
F_1_- 2	13	41
F_1_- 3	37	123
F_1_- 9	8	26
**CIM2GTAIL P1**		
F_1_- 1	4	17
F_1_- 5	2	8
F_1_- 9	8	26
**Grand total**	83	260

## Discussion

The DH technology offers the fastest route of inbred line development by rapidly fixing the desirable combinations in a single year as compared to conventional breeding programs. Many HISs were developed under temperate conditions that showed similar HIR under tropical conditions but were poor in agronomic performance and adaptability to tropical conditions [20, 23]. The differential response of haploid induction to the genetic background of maternal lines accompanied by low HIR and high mortality rate due to artificial chromosomal doubling of haploid seedlings creates a hindrance in DH production on a commercial scale under tropical conditions. In the present study, the HIR obtained from CIM2GTAIL P2 was also calculated along with CIM2GTAIL P1. The results showed that CIM2GTAIL P2 has a higher mean HIR (10%) as compared to CIM2GTAIL P1 (7.46%). The mean HIR obtained from CIM2GTAIL P1 is also higher (7.46%) than the previous reports (5.48%) [13]. On an individual basis, the HIR obtained from various crosses of CIM2GTAIL P1 and source population ranges from 6.6 to 8.5% and that of CIM2GTAIL P2 with source population ranges from 8.1 to 13.7%. These findings are relevant to the previous studies that have reported that the HIR of inducer stocks varies from 6 to 15% [5]. Also, the present research conducted has evaluated the response of CIM2GTAIL P2 in terms of HIR that has not been previously reported. The in vivo HIR is also influenced by the genotype of the inducer and the source populations [19]. Similar results have been obtained in this research work.

The HIR is calculated on the basis of the Navajo (R1-nj) phenotype. According to a report by Chaikam et al. [7], this phenotype is inhibited in about 8% of the induction crosses comprising diverse source populations. Factors such as grain structure and kernel color are also important for haploid identification. The *R1-nj* expression could be influenced by the genetic background of the female parent (source population).

The *C1* anthocyanin regulatory locus is the determining genetic factor that influences the inhibition of the Navajo phenotype [6]. The haploid identification is difficult in source populations containing dominant *C1-I*, which is mostly found in flint maize [9, 22]. Low expression was found in yellow maize, however, there is no significant difference between yellow and white maize for anthocyanin expression [6]. Therefore, constitution of the source population that is used for DH production in tropical and sub-tropical background is also a significant factor for determining the efficacy of the DH line development.

On the other hand, faithful chromosomal doubling of the haploid seedlings is an essential part of the DH breeding program. Therefore, efforts were made to optimize the protocol for successful chromosomal doubling along with good SR of the seedlings. Varied concentrations of colchicine and DMSO solutions were used for this purpose. In a study by Chaikam et al. [2], it is shown that there is no significant difference in SR and OSR at 0.07% and 0.1% of colchicine concentrations. However, in this study, it was observed that the SR increased without adverse effect on seedlings as the amount of colchicine increased from 0.04 to 0.07%. On the other hand, the mortality rate elevates when the concentration is increased to 0.1% due to the toxicity of this chemical. The doubling efficiency is highly affected by the amount of chemicals utilized.

## Conclusion

According to the findings, the genotype of the source populations and inducer stocks along with the concentrations of chemicals used are the determining factors for the efficient production of DH lines. The colchicine and DMSO concentrations of 0.07% and 0.1% respectively have been proven to be effective for the development of DH lines at a large scale. Also, the optimized protocol developed using CIMMYT haploid inducer CIM2GTAIL P2 for efficient doubled haploid production will not only fasten the breeding program but will reduce the production cost of doubled haploid with great efficiency in sub-tropical maize.

## Materials and methods

### Germplasm used

The source germplasm, the population from which DH lines are to be obtained by the use of maternal HIS, is determined based on the objective of the breeding program. Thirteen F_1_s developed from elite backgrounds of high seed yield potential dovetailed with good local adaptation were selected. CIMMYT-derived 2GTAILs i.e., CIM2GTAIL P1 and CIM2GTAIL P2 were used as pollen parents for carrying out the induction crosses. The experiments were carried out at the maize experimental fields of Punjab Agricultural University, Ludhiana, India for two seasons (Spring 2021 and *Kharif* 2021). Both the inducers carried the *R1-nj* marker which causes distinct purple coloration in the endosperm as well as the embryo that facilitate the identification of putative haploids.

### Methodology

#### Generation of induction crosses

The two haploid inducer stocks, CIM2GTAIL P1 and CIM2GTAIL P2 were used as pollen parents and crossed with 13 high potential sub-tropical adapted F_1_s to generate the induction crosses during 2021. Thirteen F_1_s were crossed with both the inducers and hence, twenty-six induction crosses were attempted. Out of 13, five F_1_s were responsive to haploid induction (Table 5) based on the expression of the Navajo phenotype. This phenotype is inhibited in about 8% of the induction crosses comprising diverse source populations [6, 7]. The populations derived from temperate x tropical/sub-tropical lines were not considered in study because very less number of DH seed was available from their induction crosses.

**Table 5:** The pedigree of F_1_s responsive to haploid induction

S. No.	F_1_s	Pedigree	Grain texture	Kernel color
1.	F_1_-1	PML-118 x PML-164	Dent	Light yellow
2.	F_1_-2	PML-114 x PML-81	Flint	Orange yellow
3.	F_1_-3	PML-119 x PML-185	Semi-dent	Yellow
4.	F_1_-5	PML-97 x PML-150	Semi-flint	Orange
5.	F_1_-9	PML-95 x PML-172	Semi-dent	Yellow

### Haploid identification

The ear of each plant was harvested separately, and the haploid seeds were sorted out manually based on the *R1-nj* marker expression [17]. The diploid seeds carry purple pigmentation on both endosperm and embryo while the haploid seeds carry pigmentation on the endosperm with no coloration on the embryo. Selfed/out-crossed seeds showed no anthocyanin coloration on both endosperm and embryo.

### Sterilization of the putative haploid seeds

The sorted putative haploid seeds were first washed with distilled water two to three times thoroughly. The seeds were then treated with 70% ethyl alcohol for 5 min. The treated seeds are washed with distilled water 2–3 times and dipped in fungicide solution (2gm/liter) for 10 min to avoid any fungal contamination. After giving 2–3 washings with distilled water, the seeds were kept for germination.

### Chromosomal doubling treatments

#### 3–4 Day old seedling treatment (T1)

In this method, the haploid seeds were germinated on germination paper for 3–4 days at 25–28ºC. The tip of the shoot and root of the germinated seedlings were cut up to 1 and 2 cm, respectively, and immersed in 0.04% colchicine solution containing 0.5% DMSO for about 12 h [4].

#### V_2_ stage seedling treatment (T2)

The haploid seeds were germinated in plastic trays in a glasshouse. The seedlings were grown for 10–12 days until they reach two-leaf stage, described as the V_2_ stage in the text. Further, the seedlings were removed from the trays carefully and their roots were washed to remove the growth mixture. The colchicine treatment is required specifically to the crown region; therefore the washed seedlings were aligned at seed level and dipped in a plastic container containing colchicine solution and DMSO for 5–6 h [2]. The procedure does not include any incision to any part of the seedling during the process. The seedlings were handled very carefully so that no damage is caused to the rootlets.

In this experiment, the different concentrations of colchicine have been used for the chromosomal doubling of putative haploids (Table 6).

**Table 6:** Different doses of colchicine and DMSO for chromosomal doubling of putative haploid seedlings

S. No.	Colchicine concentration (%)	DMSO concentration (%)
**3–4 day old seedling treatment (T1)**		
1.	0.04	0.5
**V**_**2**_**stage treatment (T2)**		
2.	0.04	0.5
3.	0.07	0.1
4.	0.10	0.1

After the treatments, the seedlings were gently rinsed and kept under running water for 30 min and the water was emptied into a plastic container for disposal. Later, seedlings were washed three to four times with tap water. Seedlings were replanted in trays and maintained for another 8 days for recovery. The colchicine solution used during the experiments was collected in a plastic container for safe disposal. Colchicine is a hazardous chemical and much more toxic than the N_2_O gas and other anti-mitotic herbicides [2]. It prevents the formation of microtubules during cell division that leads to duplication of the chromosomal number in the cell [15]. In humans, it impairs the protein assembly and affects the cellular functions leading to the multi-organ dysfunction and failure [10]. Therefore, it requires safe disposal after use. After recovery, seedlings were transplanted in a well-irrigated field provided with good agronomic management.

After 3–4 weeks, the plants were screened in the field based on their phenotypic characteristics such as plant vigor and erectness of leagves. The false positives were rogued out and comparatively weak plants with erect leaves were raised [3].

### Selfing of fertile D_0_ plants

**Fig. 4:**
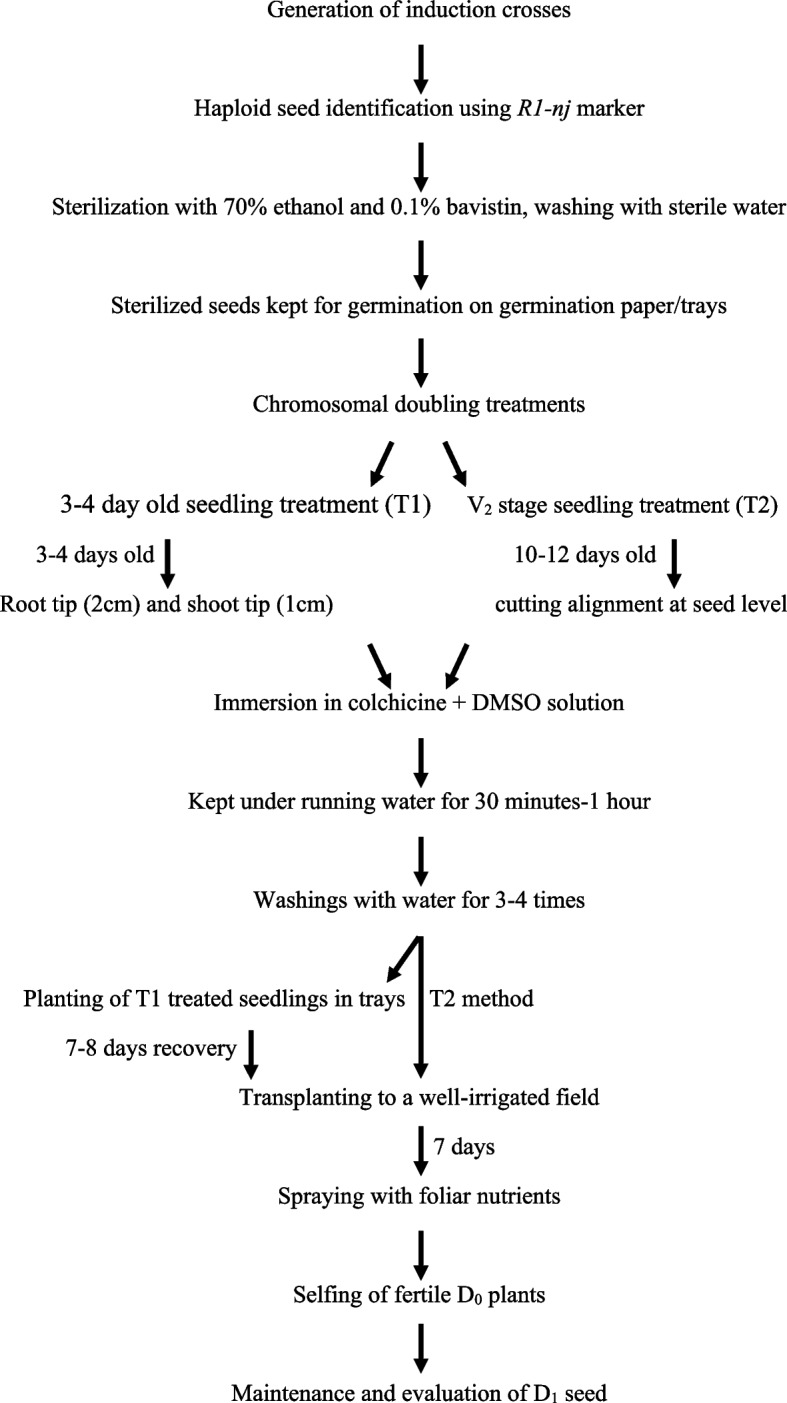
Flow diagram depicting the workflow of the experiment conducted

**Fig. 5:**
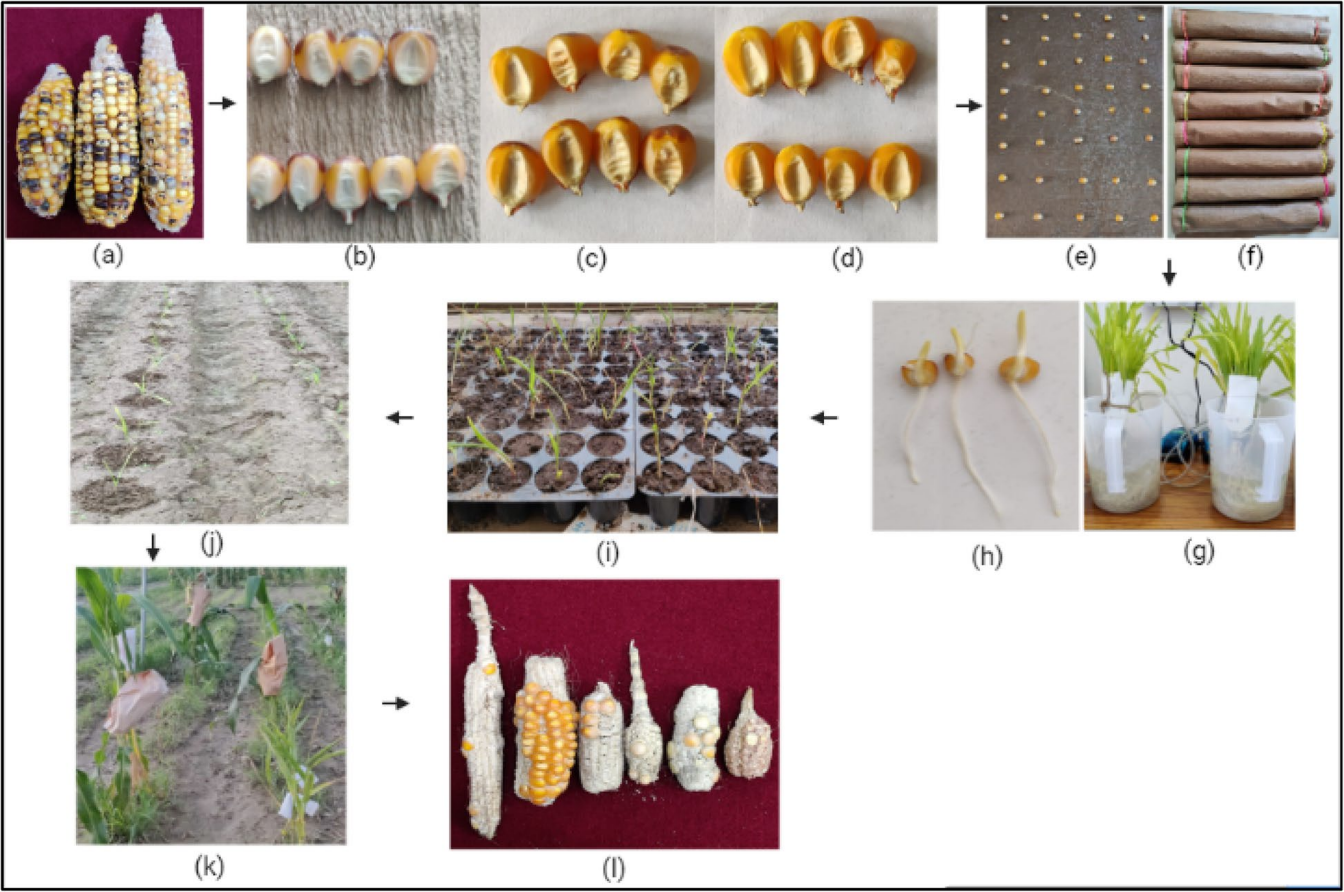
**a **Cobs obtained after induction crosses; **b** Putative haploid seeds; **c** F1 seeds; **d** Selfed/out-crossed seeds; **e** and **f** Germination of putative haploid seeds; **g** Colchicine treatment at V_2_ (T2) and **h** at 3–4 day old seedling stage (T1); **i** Hardening of treated seedlings; **j** Seedlings transferred to field; **k** Selfing of D_0_ plants; **l** Cobs showing DH seeds obtained after selfing

The fertile D_0_ plants were identified at the time of anthesis. The ears of the fertile D_0_ plants were covered with butter paper bags before silk emergence. The tassel bags were used to cover the tassels for pollen collection. Care was taken not to cause any damage or tassel breakage. The pollen was collected the next morning and shed on the ear of the same plant and the same tassel bag was used for covering the ear. Plants were raised to maturity and monitored regularly. The ears were harvested after 40–45 days of pollination. The D_1_ seeds obtained after self-pollination were subsequently raised for maintenance and agronomic evaluation. The flow chart of the whole procedure followed to raise DH is depicted in Figs. 4 and 5.

### Data collection

The following data were recorded in the experiment: (1) total number of seeds obtained from induction crosses; (2) number of putative haploid seeds sorted; (3) number of seeds germinated; (4) number of seeds/seedlings subjected to treatment; (5) number of D_0_ plants survived; (6) number of D_0_ plants that produced seed.

The HIR, SR and OSR were calculated as follows and expressed in percentage:

$$HIR=\frac{No.\;of\;putative\;haploid\;seeds}{Total\;no.\;of\;seeds\;obtained\;from\;induction\;cross}$$

$$SR=\frac{No.\;of\;plants\;survived}{No.\;of\;seedlings\;subjected\;to\;treatment}$$

$$OSR=\frac{No.\;of\;Do\;plants\;that\;produced\;seed}{No.\;of\;seedlings\;subjected\;to\;treatment}$$

## Data Availability

All data generated or analyzed in this study are included in this published article.

## Abbreviations

DH: Doubled haploid
CIM2GTAILs: CIMMYT derived second generation haploid inducers
HI: Haploid induction
HIS: Haploid inducer stock
HIR: Haploid induction rate
SR: Survival rate
OSR: Overall success rate
D_0_: Doubled haploid population obtained after treatment of haploid seedlings
D_1_: First generation of doubled haploid population
